# An Umbrella Review of Meta-Analyses on Transcrestal Sinus Floor Elevation: Methodological Quality, Primary-Study Overlap, and Interpretive Boundaries

**DOI:** 10.3390/dj14080483

**Published:** 2026-08-05

**Authors:** Hadar Better, Keren Better, Thabet Asbi, Shaul Lin, Gavriel Chaushu, Doron Haim

**Affiliations:** 1 Maccabi-Dent Medical & Quality Assurance Department, Tel Aviv 6801298, Israel; 2 Maccabi-Dent Research and Innovation Department, Tel Aviv 6801298, Israel; 3 Department of Information and Computing Sciences, Utrecht University, 3584 CC Utrecht, The Netherlands; 4 Department of Periodontology, Rambam Health Care Campus, Haifa 3109601, Israel; 5 Department of Oral and Maxillofacial Surgery, Rabin Medical Center, Petach Tikva 4941492, Israel; 6 Department of Endodontics and Dental Trauma, Rambam Health Care Campus, Haifa 3109601, Israel; 7 The Ruth and Bruce Rappaport Faculty of Medicine, Technion—Israel Institute of Technology, Haifa 3525422, Israel; 8 Department of Health Systems Management, Faculty of Health Sciences, Ariel University, Ariel 40700, Israel

**Keywords:** umbrella review, meta-analysis, transcrestal sinus floor elevation, AMSTAR 2, biomechanical class, Corrected Covered Area, methodological quality, Schneiderian membrane perforation, benign paroxysmal positional vertigo, patient-reported outcomes

## Abstract

**Background/Objectives**: Transcrestal sinus floor elevation (TSFE) includes several surgical approaches that differ in access, instruments, and force delivery. Existing meta-analyses often combine these approaches. This umbrella review examined methodological quality, primary-study overlap, and the limits of clinical interpretation across three decisions: short implants versus sinus floor elevation, transcrestal versus lateral access, and technique choice within transcrestal elevation. **Methods**: Following the Cochrane Handbook and PRISMA 2020 reporting standards, eligible meta-analyses on TSFE published between 2015 and April 2026 were identified through a three-phase systematic search across PubMed, EMBASE, Cochrane CENTRAL, Web of Science, and Scopus. Methodological quality was assessed using AMSTAR 2 with verbatim per-item evidence; primary-study overlap was quantified using the Corrected Covered Area. **Results**: Thirty meta-analyses met the inclusion criteria. AMSTAR 2 confidence was MODERATE in nine reviews, LOW in 11, and CRITICALLY LOW in 10; none was rated HIGH. Pool-level Corrected Covered Area was 0.92%, while cluster-level overlap reached 10.90% in the graftless osteotome cluster. Implant survival was generally high across the reviewed comparisons. Data on membrane perforation, benign paroxysmal positional vertigo (BPPV), and patient-reported outcomes were less complete and were reported inconsistently. Within the limited technique-specific evidence, BPPV was reported mainly with osteotome-based procedures, including one case among 24 patients (4.17%) in the osteotome group reported by Cobo-Vázquez et al. **Conclusions:** The available meta-analytic evidence supports high implant survival across the main treatment options, but it does not support a simple ranking of techniques. Clinical interpretation should follow the decision being made: whether sinus elevation is needed, whether transcrestal or lateral access is appropriate, and which transcrestal technique fits the case. Signals for BPPV and other complications with osteotome-based procedures are suggestive, but event reporting is sparse and certainty is limited. Biomechanical explanations should therefore be treated as clinical context rather than as causal findings of this umbrella review.

## 1. Introduction

Transcrestal sinus floor elevation (TSFE) consistently shows survival rates exceeding 95% across multiple systematic reviews and meta-analyses. This remarkable consistency obscures a critical clinical question: what techniques are we actually pooling under this anatomical umbrella?

Most meta-analyses group these approaches under one TSFE label or divide them by graft use, implant length, or timing. Few separate techniques by how force is delivered. Because survival is generally high, complications and patient-reported outcomes may help distinguish options, but these outcomes are reported less consistently.

Osteotome-mediated, drill-based, hydraulic, piezoelectric, and osseodensification approaches use the same anatomical access but differ in instruments and force delivery. These differences may help explain some reported complication patterns. They do not, however, establish a causal hierarchy among techniques.

This umbrella review synthesises the available meta-analytic evidence on TSFE using the Cochrane Handbook for Systematic Reviews of Interventions (Chapter V) [1]. It combines methodological-quality assessment with primary-study overlap analysis. The review addresses three clinical decisions: short implants versus sinus floor elevation, transcrestal versus lateral access, and technique choice within transcrestal elevation. Biomechanical classification was used only as an interpretive framework for the third decision.

## 2. Materials and Methods

### 2.1. Study Design and Reporting Standards

This umbrella review of meta-analyses on TSFE was conducted in accordance with the Cochrane Handbook for Systematic Reviews of Interventions (Chapter V) [1] and the JBI Manual for Evidence Synthesis [2]. The review is reported following PRISMA 2020 [3], with adaptations for overviews of reviews. The review was not prospectively registered in PROSPERO because the umbrella-review protocol was finalised after screening had begun. This is reported as a limitation. To support reproducibility, the Zenodo package provides the search strategy, study-selection summary, included-review list, AMSTAR 2 ratings, Corrected Covered Area results and heatmap, source-checked complication and PROM summaries, technique mapping, and methodological decision notes (https://doi.org/10.5281/zenodo.21432230, accessed on 18 July 2026) under a CC-BY 4.0 licence.(Supplementary Table S1).

### 2.2. Eligibility Criteria

A PICO-S framework defined the eligibility criteria before final study selection and data extraction (the eligibility criteria were documented in the protocol file deposited at Zenodo; this review was not prospectively registered in PROSPERO for the reasons explained in Section 4.6).

Population: Partially or fully edentulous adult patients undergoing dental implant placement in the posterior maxilla with insufficient residual bone height (RBH) requiring sinus floor elevation.

Intervention: Transcrestal sinus floor elevation techniques, including osteotome (manual and screwable), osseodensification, hydraulic, ultrasonic, and rotary approaches.

Comparison: Any meaningful comparator employed within the included meta-analyses, including alternative TSFE techniques, lateral sinus floor elevation, and short implants placed without sinus elevation.

Outcomes: Implant survival rate (primary); marginal bone loss, endosinus bone gain, Schneiderian membrane perforation rate, intra- and post-operative complications including benign paroxysmal positional vertigo, and patient-reported outcome measures (secondary).

Study design (S): Systematic reviews with quantitative meta-analysis (pairwise, network, or single-arm). Narrative reviews and systematic reviews without quantitative synthesis were excluded.

Additional inclusion criteria: publication in English between 2015 and April 2026; reporting of at least one pooled estimate with confidence interval, heterogeneity statistic, or forest plot. Exclusion criteria: paediatric, animal, or in vitro populations; conference abstracts and editorials; meta-analyses limited to lateral sinus floor elevation without a transcrestal arm; duplicate publications.

Meta-analyses were included only when they contained a transcrestal sinus floor elevation arm, a transcrestal subgroup, or a comparator structure directly informing the choice between TSFE and clinically relevant alternatives (lateral sinus floor elevation, short implants placed without elevation, or graftless transcrestal protocols). Reviews on lateral sinus floor elevation only without any transcrestal comparator were excluded; reviews on short implants were included only when sinus floor elevation served as the explicit comparator.

### 2.3. Information Sources and Search Strategy

The literature search was conducted in three phases across five databases. Eligible publications were restricted to meta-analyses published between January 2015 and the respective search dates (rationale provided in Section 2.2). Phase 1 used PubMed/MEDLINE, with iterative searches conducted through 2025 and a final update in December 2025; the search applied controlled vocabulary (MeSH terms) and free-text title/abstract terms for the intervention and publication-type components, with no further publication-year restriction (the 2015 lower bound was applied at screening). The full Boolean string was: (“sinus floor elevation” [Title/Abstract] OR “sinus floor augmentation” [Title/Abstract] OR “sinus lift” [Title/Abstract] OR “maxillary sinus floor” [Title/Abstract] OR “transcrestal sinus” [Title/Abstract] OR “osteotome sinus” [Title/Abstract] OR “OSFE” [Title/Abstract] OR “TSFE” [Title/Abstract] OR “TSFA” [Title/Abstract] OR “LSFE” [Title/Abstract] OR “LSFA” [Title/Abstract] OR “osseodensification” [Title/Abstract]) AND (“meta-analysis” [Title/Abstract] OR “systematic review” [Title/Abstract] OR “umbrella review” [Title/Abstract] OR “network meta-analysis” [Title/Abstract]).

Phase 2 was conducted on 22 April 2026 across three databases, with each search applying a 2015–2026 publication-year filter: EMBASE (via Embase.com) using Emtree terms with free-text equivalents; Cochrane CENTRAL through the Cochrane Library Advanced Search using Title/Abstract/Keyword field tags; and Web of Science Core Collection using TS = field tags with the Review Article document-type filter. Database-specific search strings, full record exports, and per-record screening decisions are deposited at Zenodo. Phase 3 was conducted on 26 April 2026 in Scopus to verify the completeness of the four-database Phase 1 + 2 strategy. The Scopus query mirrored the Phase 2 Boolean structure and returned 269 records. Triage proceeded by three sequential steps: DOI-matching against the existing pool (which confirmed Scopus indexing of 22 of 28 prior pool meta-analyses in the Phase 1 + 2 reference set), title/abstract filtering of remaining records, and full-text assessment of triage candidates. Phase 3 yielded two additional eligible meta-analyses (Vetromilla 2021 [4] and Toledano 2022 [5]).

Reference lists of included meta-analyses and recent narrative reviews on TSFE were hand-searched; no additional unique meta-analyses beyond those identified by database searches were retrieved.

### 2.4. Selection Process

**Figure 1:**
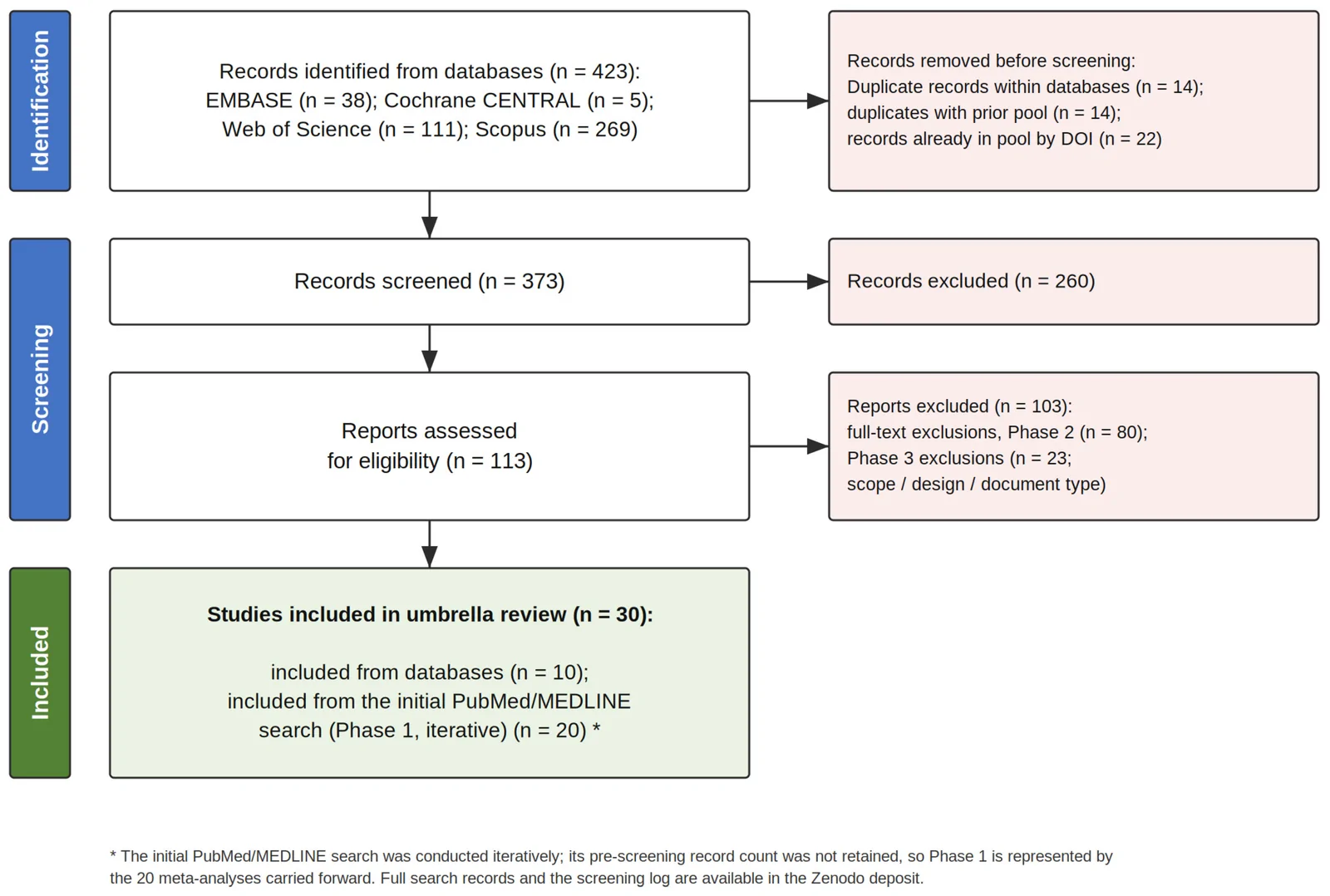
PRISMA 2020 flow diagram of the three-phase search strategy. Phase 1 (PubMed/MEDLINE) was conducted iteratively through 2025 with the final update in December 2025; Phase 2 (EMBASE, Cochrane CENTRAL, Web of Science) was conducted on 22 April 2026; Phase 3 (Scopus supplementary verification) was conducted on 26 April 2026. Cross-database deduplication was performed in two passes for Phase 2 (within-Phase 2 and Phase 2 versus Phase 1) and a third pass for Phase 3 (Scopus versus the Phase 1 + 2 pool). Records excluded at full-text assessment had documented reasons recorded in the screening log deposited at Zenodo. Hand-searching of reference lists from included meta-analyses and recent narrative reviews on TSFE identified no additional unique meta-analyses. The two meta-analyses identified in Phase 3 were Vetromilla et al. [4] and Toledano et al. [5].

Records identified in Phase 2 and Phase 3 were combined and processed in three stages. First, deduplication was performed across passes: for Phase 2, internal deduplication across the three supplementary databases (records indexed in two or more sources were collapsed) was followed by removal of records already identified in Phase 1; for Phase 3, DOI-matching against the existing Phase 1 + 2 pool confirmed Scopus indexing of 22 of the 28 prior pool meta-analyses, and the remaining 247 records were screened de novo. Second, titles and abstracts of remaining records were screened against the eligibility criteria; records clearly outside the scope of the umbrella review were excluded with documented reasons. Third, full-text assessment was conducted for all records that passed title and abstract screening, applying all eligibility criteria. Each excluded full-text record was assigned a documented reason. The final pool comprised meta-analyses identified across all three phases. The selection process is summarised in the PRISMA 2020 [2] flow diagram (Figure 1). Per-record decisions, exclusion reasons, and full-text assessment outcomes are preserved in the screening log deposited at Zenodo.

### 2.5. Data Extraction

A structured extraction form was prepared before final extraction and applied to all included meta-analyses. The form covered bibliographic details, review methods, PICO elements, pooled outcomes, complications, patient-reported outcomes, and technique classification. H.B. completed the first clinical and methodological extraction. K.B. independently checked all included records, all numerical values used in the manuscript, and all technique assignments against the full-text sources. Differences were resolved by discussion and recorded in the decision log. This was a verified two-reviewer process, but it was not a fully independent duplicate extraction of every field. The same approach was used for the final AMSTAR 2 ratings: one reviewer completed the first rating and the second reviewer independently checked the supporting text and final tier. Verification files and decision-log entries are available at Zenodo.

### 2.6. Methodological Quality Assessment

The methodological quality of each included meta-analysis was assessed using AMSTAR 2 [6], which contains 16 items and seven critical items (2, 4, 7, 9, 11, 13, and 15). Each rating was linked to verbatim supporting text from the source review. Overall confidence followed the AMSTAR 2 guidance: HIGH, MODERATE, LOW, or CRITICALLY LOW according to the number and type of weaknesses. A second reviewer independently checked the supporting text and the final tier. Any change after the second check was recorded in the decision log. The full item-level ratings for all 30 reviews are available at Zenodo. Table A1 provides examples from five reviews and shows how the critical items were judged.

AMSTAR 2 [6] was used as a domain-based critical appraisal tool rather than as a numerical quality score; therefore, synthesis was stratified by confidence tier and by the presence of critical weaknesses rather than by summed item counts.

### 2.7. Primary-Study Overlap (Corrected Covered Area)

Primary-study overlap across the 30 included meta-analyses was quantified using the Corrected Covered Area methodology (Pieper et al., 2014) [7]. For each meta-analysis, the complete list of primary studies was extracted and matched across the pool using normalised author–year–title fingerprints; matches were verified by DOI or PMID where available, and ambiguous matches were resolved by full-citation comparison. CCA was computed at the pool level using the formula CCA = (N − r)/(rc − r), where N is the total number of primary-study inclusions across all meta-analyses, r is the number of unique primary studies, and c is the number of meta-analyses. Pieper et al. (2014) [7] interpretive thresholds were applied: slight overlap (CCA 0% to <5%), moderate overlap (5% to <10%), high overlap (10% to <15%), and very high overlap (≥15%).

Cluster-level CCA was additionally computed for thematically coherent groups of meta-analyses (graftless osteotome cluster; short-implant-versus-SFE cluster; perforation–survival cluster; LSFE long-term cluster; technique-comparison cluster) to reveal overlap patterns that the pool-level estimate may obscure when meta-analyses address heterogeneous comparator structures. Cluster definitions and the full overlap matrix are deposited at Zenodo.

### 2.8. Synthesis Approach

The included meta-analyses differed in populations, comparators, outcome definitions, follow-up, and sets of primary studies. Re-pooling their pooled estimates would have counted some primary studies more than once and would have combined clinically different questions. We therefore used narrative synthesis. Results were kept within three clinical questions: short implants versus sinus floor elevation, transcrestal versus lateral access, and comparisons among transcrestal techniques. Quantitative estimates are reported as presented in the original meta-analyses. No new pooled effect was calculated. AMSTAR 2 confidence and cluster-level overlap were used to judge how much weight to place on each result.

## 3. Results

### 3.1. Search Results and Pool Composition

The three-phase systematic search yielded 30 meta-analyses meeting all eligibility criteria. Phase 1 (PubMed/MEDLINE, with the final update conducted in December 2025) identified 20 meta-analyses through searching with controlled vocabulary and free-text terms. Phase 2 (EMBASE, Cochrane CENTRAL, Web of Science; 22 April 2026) returned 154 records (EMBASE = 38; Cochrane CENTRAL = 5; Web of Science = 111). Internal deduplication across the three Phase 2 databases removed 14 records appearing in two or more sources, and a second deduplication pass against the Phase 1 pool removed an additional 14 records, leaving 126 records eligible for screening. Title/abstract screening excluded 38 records as outside the umbrella-review scope, and full-text assessment excluded a further 80 records with documented reasons. Phase 3 (Scopus supplementary verification, 26 April 2026) returned 269 records: DOI-matching against the existing pool confirmed Scopus indexing of 22 of 28 prior pool meta-analyses (Phase 1 + 2 reference set); 25 records were retained as candidates after non-meta-analysis (n = 54) and out-of-scope (n = 168) filtering; 10 candidates entered full-text triage; two were eligible per Section 2.2 (Vetromilla 2021 [4] and Toledano 2022 [5]) and eight were excluded with documented reasons (seven on scope/design grounds and one on document-type grounds, namely Merli et al. 2018 [8], an umbrella review of systematic reviews that did not perform quantitative re-pooling; by contrast, Vetromilla 2021 [4] was retained because, although structured as an umbrella review of meta-analyses, it performs quantitative re-pooling of risk ratios across its component reviews and reports pooled estimates with confidence intervals, thereby meeting the eligibility criterion of “systematic reviews with quantitative meta-analysis” defined in Section 2.2). The final pool comprised 30 meta-analyses (Phase 1: 20; Phase 2: 8; Phase 3: 2). Hand-searching of reference lists from included meta-analyses and recent narrative reviews on TSFE identified no additional unique meta-analyses. The complete selection process is summarised in Figure 1 (PRISMA 2020 [3] flow diagram).

The full pool of 30 included meta-analyses, with bibliographic details, comparator structures, primary outcomes, and AMSTAR 2 confidence tiers, is presented in Table 1.

### 3.2. Methodological Quality (Results)

AMSTAR 2 [6] assessment of the 30 included meta-analyses yielded the following confidence distribution: no meta-analysis achieved HIGH confidence; nine (30.0%) were rated MODERATE; 11 (36.7%) were rated LOW; and 10 (33.3%) were rated CRITICALLY LOW. The MODERATE-tier subset comprised Ravidà 2019 [16], Lee 2023 [22], Shi S 2023 [24], Kadkhodazadeh 2024 [25], Moraschini 2017 [31], Mester 2023 [33], Yan 2019 [34], Raghoebar 2019 [36], and Toledano 2022 [5]. Critical flaws driving CRITICALLY LOW ratings most frequently involved Item 2 (a priori protocol/registration), Item 7 (list of excluded studies with justification), and Item 15 (publication-bias assessment), often in combination.

During second-pass verbatim re-rating, nine formal tier revisions were documented. In addition, three meta-analyses with previously provisional summary-level entries in Table 1 were corrected to align with their final per-item AMSTAR 2 assessments: Geminiani 2017 [10], Potdukhe 2025 [23], and Wang M 2022 [35] (provisional LOW → confirmed CRITICALLY LOW after verbatim per-item re-rating identified additional critical flaws). Five meta-analyses were downgraded: Al-Moraissi 2018 [12] perforation (MODERATE → CRITICALLY LOW; two-step downgrade for absent a priori protocol and aggregate-only excluded-studies reporting), Antonoglou 2018 [14] (MODERATE → LOW; Item 7 incompleteness), Al-Moraissi 2019 [15] NMA (MODERATE → LOW; Item 7 incompleteness), Starch-Jensen 2025 [27] (MODERATE → LOW; single-reviewer signature on Item 9), and Menini 2025 [28] (MODERATE → LOW; aggregate PRISMA flow on Item 7). Two meta-analyses were upgraded: Ravidà 2019 [16] (LOW → MODERATE; all five critical differentiator items confirmed Yes) and Kadkhodazadeh 2024 [25] (LOW → MODERATE; PROSPERO registration with RoB 2 and GRADE confirmed verbatim). Two meta-analyses were further downgraded from LOW to CRITICALLY LOW: Guo 2020 [17] (two critical flaws confirmed) and Lin 2021 [18] (Items 9 and 13 failures confirmed). Phase 3 verification added two further meta-analyses with verbatim AMSTAR 2 assessment: Vetromilla 2021 [4] (LOW; one critical flaw on Item 15: no publication-bias assessment with no <10-studies justification, per the precedent established with Antonoglou 2018 [14] and Al-Moraissi 2019 [15]) and Toledano 2022 [5] (MODERATE; zero critical flaws; PROSPERO 295642, RoB 2 with funnel plots, and reference-list hand-searching across four databases). Per-item ratings, supporting verbatim quotations, and tier-revision rationales for all 30 meta-analyses are deposited at Zenodo.

### 3.3. Primary-Study Overlap Analysis

Pool-level Corrected Covered Area across the 30 included meta-analyses was 0.92%, classifying as slight overlap per the Pieper et al. 2014 [7] thresholds (slight: 0% to <5%; moderate: 5% to <10%; high: 10% to <15%; very high: ≥15%). At cluster level, however, overlap was substantially higher within thematically focused subsets. The graftless osteotome-mediated TSFE cluster (seven meta-analyses: Moraschini 2017 [31], Rahate 2023 [30], Chen and Shi 2018 [11], Duan 2017 [9], Guo 2020 [17], Ye 2021 [19], Shi QH 2022 [21]) yielded a cluster-level CCA of 10.90% (high overlap), driven by a small set of repeatedly re-analysed RCTs, notably Nedir 2013/2016 and Si 2013/2016, that appeared in five or more of the seven cluster meta-analyses. The short-implant-versus-SFE cluster (eight meta-analyses with the Phase 3 additions: Ravidà 2019 [16], Lin 2021 [18], Tang 2022 [20], Mester 2023 [33], Yan 2019 [34], Wang M 2022 [35], Vetromilla 2021 [4], and Toledano 2022 [5]) yielded a cluster-level CCA of 6.8% (moderate overlap), with the increase relative to the pre-Phase-3 pool of 28 meta-analyses driven by the introduction of Toledano 2022’s 14 RCTs (Magdy 2021, Shi J-Y 2021, Rossi 2021, Nielsen 2021, Esposito 2019, Felice 2019 a+b, Guljé 2019, Thoma 2018, Bolle 2018, Bechara 2017, Gastaldi 2017, Shi J-Y 2019, Schincaglia 2015), of which several were already represented within the cluster’s primary-study substrate via Mester 2023 [33] and Yan 2019 [34]. The three remaining pre-specified clusters were characterised descriptively rather than with a single numeric CCA value, because each draws on a largely independent primary-study substrate with limited within-cluster re-analysis such that pooled CCA computation would not meaningfully summarise overlap structure. The perforation–survival cluster comprises Al-Moraissi 2018 [12], Schiavo-Di Flaviano 2024 [29], and Lee 2023 [22]; the LSFE long-term cluster comprises Raghoebar 2019 [36], Antonoglou 2018 [14], and Aludden 2018 [13]; the technique-heterogeneity cluster comprises Geminiani 2017 [10], Al-Moraissi 2019 [15], Shah 2022 [32], Kadkhodazadeh 2024 [25], Cobo-Vázquez 2025 [26], Potdukhe 2025 [23], Shi S 2023 [24], and Menini 2025 [28]. The complete CCA matrix, with citation pair counts and per-cluster computations, is deposited at Zenodo.

### 3.4. Implant Survival Outcomes

Implant survival was reported across all 30 included meta-analyses, with pooled or descriptive estimates consistently within the 95–100% range across the entire spectrum of TSFE techniques, comparators, and follow-up windows. Anchoring on the MODERATE-tier subset, Moraschini 2017 [31] reported a cumulative pooled implant survival rate of 97% across 1034 implants placed in transcrestal sinus floor elevation without grafting material, with no significant difference in survival between grafted and graftless approaches (RR 0.55, 95% CI 0.26–1.19; *p* = 0.13). Raghoebar 2019 [36] reported a cumulative weighted annual implant loss of 0.43% (95% CI 0.38–0.49%) for lateral SFE, translating to 97.8% 5-year survival, with no significant difference between simultaneous and delayed placement or between autogenous bone and bone substitutes. Shi S 2023 [24] reported survival equivalence between TSFA and LSFA at residual bone heights ≤6 mm (RR ≈ 1.0). Across the short-implant-versus-SFE subgroup (Ravidà 2019 [16], Mester 2023 [33], Yan 2019 [34], and the Phase 3 additions Vetromilla 2021 [4] and Toledano 2022 [5]), pooled survival risk ratios converged toward unity (RR 0.97–1.08; all non-significant). Vetromilla 2021 [4] (umbrella review of 7 SRMAs; LOW tier) reported pooled RR 1.08 (95% CI 0.61–1.90; *p* = 0.79; I^2^ = 0%) across five component meta-analyses; Toledano 2022 [5] (14 RCTs; MODERATE tier) reported pooled RR 1.02 (95% CI 1.00–1.05; *p* = 0.09; I^2^ = 0%) across 12 component RCTs. The convergence holds whether implant length is anchored at ≤8 mm (Vetromilla threshold) or ≤6 mm (Toledano threshold), indicating that implant length within the 6–10 mm range did not materially alter survival within the reviewed meta-analytic evidence. The LOW and CRITICALLY LOW tier meta-analyses reported survival values within the same 95–100% band, with no meta-analysis identifying a technique with materially divergent survival. The two short-implant-versus-SFE reviews added through Phase 3 (Vetromilla 2021 [4] and Toledano 2022 [5]) inform the decision boundary between sinus floor elevation and non-elevation alternatives; they were not used to infer superiority among TSFE techniques. A conceptual sensitivity check confirmed that excluding Vetromilla 2021 [4] would not alter the direction of the short-implant-versus-SFE inference, because the same conclusion is supported by RCT-based meta-analyses retained in the pool independently of Vetromilla (Ravidà 2019 [16], Yan 2019 [34], Mester 2023 [33], and Toledano 2022 [5]); the Vetromilla 2021 [4] re-pooled estimates contribute corroborative rather than load-bearing evidence.

### 3.5. Membrane Perforation

Membrane perforation was reported as a primary or secondary outcome in eleven of the 30 included meta-analyses. Pooled and descriptive estimates spanned a wide range, reflecting the heterogeneity of the underlying primary studies and the variability of perforation case definitions across reports. Within the MODERATE-tier subset, three meta-analyses provided directly comparable estimates: Shi S 2023 [24] reported a risk ratio of 0.71 favouring transcrestal over lateral approaches at residual bone height ≤6 mm (95% CI included unity; non-significant trend); Lee 2023 [22] (Bayesian network meta-analysis) ranked the six included techniques by Surface Under the Cumulative Ranking Curve, with TSFE-reamer attaining the highest rank (SUCRA 0.94, lowest perforation probability) and lateral-window-rotary attaining the lowest rank (SUCRA 0.07, highest perforation probability); Yan 2019 [34] reported a perforation/infection risk ratio of 0.11 (95% CI 0.02–0.63; *p* = 0.01) favouring short implants placed without elevation over the sinus elevation comparator. Within the broader pool, Ye 2021 [19] reported a 3.08% perforation rate in non-grafted osteotome procedures; the pattern of lower perforation rates in transcrestal versus lateral approaches was consistent across reports, with transcrestal approaches typically reporting single-digit perforation rates compared with the higher rates characteristic of lateral-window procedures in the underlying primary literature.

### 3.6. Benign Paroxysmal Positional Vertigo

Benign paroxysmal positional vertigo (BPPV) was reported in four meta-analyses, with a suggestive technique-related signal: BPPV occurrences were concentrated in studies using mallet-driven osteotome techniques (impact class), and were rare or absent in studies using drill-based, screwable, hydraulic, or piezoelectric techniques (non-impact classes). Anchoring on MODERATE-tier evidence, Kadkhodazadeh 2024 [25] reported BPPV in four of seventeen included randomised controlled trials, with three of four using osteotomes; in the Sammartino 2010 trial, as reported within Kadkhodazadeh 2024 [25], all three sinus lining perforation cases occurred in the mallet-osteotome arm with none in the screwable-osteotome arm; this same trial was also among the three osteotome studies reporting BPPV. The authors of Kadkhodazadeh 2024 [25] explicitly concluded that caution should be taken when using hand osteotomes because of higher rates of sinus lining perforation and reported patient vertigo.

Starch-Jensen 2025 [27] reported zero BPPV cases in transcrestal modified expansion-osteotomy with drilling (TSMEOD; non-impact) versus one to five cases in conventional osteotome-mediated sinus floor elevation (OMSFE; impact), with a between-group difference in *p* = 0.002. Ye 2021 [19] and Cobo-Vázquez 2025 [26] corroborated the low frequency of postoperative vertigo in non-impact transcrestal subgroups; Cobo-Vázquez 2025 [26] reported overall complication rates of 2.78% for osseodensification versus 14.32% for osteotome approaches, with BPPV cases (4.17%) restricted to the osteotome subgroup, and Ye 2021 [19] characterised vertigo as “less frequent” as a qualitative descriptor.

### 3.7. Patient-Reported Outcomes

Patient-reported outcomes were the main focus of only one included meta-analysis (Menini 2025 [28], LOW AMSTAR 2 confidence) and appeared as secondary outcomes in several others. The measures varied widely, including pain scales, oral-health quality-of-life measures, swelling, analgesic use, and willingness to repeat treatment. The available reports generally favoured less invasive procedures, but the evidence was sparse and could not support firm comparisons among individual transcrestal techniques. A structured summary of PROM availability, instruments, and limitations is provided in the Zenodo supplement.

### 3.8. Biomechanical-Class Synthesis

Technique-specific evidence was limited and uneven. Implant survival was generally high across osteotome, drill-based, hydraulic, piezoelectric, and osseodensification approaches. Some reviews reported more BPPV, discomfort, or other complications with osteotome-based procedures. For example, Cobo-Vázquez 2025 [26] reported one BPPV case among 24 osteotome-treated patients (4.17%), and Starch-Jensen 2025 [27] reported a significant difference in BPPV reporting between the compared approaches (*p* = 0.002). These findings are signals, not proof of causation. The number of events was small, outcome definitions varied, and many contributing reviews had LOW or CRITICALLY LOW AMSTAR 2 confidence. Table 2 therefore presents technique classes, operational criteria, and the limits of the available evidence without ranking the non-impact subclasses.

Table 2 summarises the operational technique classes and the evidence available for survival, complications, and patient-reported outcomes. The table is descriptive and does not imply a causal ranking.

### 3.9. Evidence Summary and Main Limitations

Table 3 summarises the main outcomes, the stronger AMSTAR 2 anchors, the consistency of findings, overlap concerns, and the main limits on interpretation. We removed the earlier adapted GRADE labels because this umbrella review did not apply a formal GRADE process at a primary-study level. The table is a descriptive evidence map and should not be read as a formal certainty rating.

AMSTAR 2 describes the quality of each included review. It does not by itself establish certainty for a clinical outcome. For that reason, Table 3 keeps the methodological signals separate and states the main limitation for each conclusion rather than assigning a new certainty category.

## 4. Discussion

### 4.1. Principal Findings

The findings are best understood as three separate clinical questions. First, meta-analyses comparing short implants with standard implants plus sinus floor elevation generally found similar survival, with different burdens of surgery and complications. Second, comparisons of transcrestal and lateral access address access-related morbidity and perforation, not the mechanism used within the transcrestal approach. Third, evidence comparing individual transcrestal techniques is limited. Osteotome-based procedures show a suggestive signal for BPPV and discomfort, but the event counts are small and do not establish causation. Across all three questions, no included meta-analysis showed a clear survival advantage for one technique. The methodological base also remains limited: no review achieved HIGH AMSTAR 2 confidence.

### 4.2. Biomechanical Framework

Biomechanical concepts may help clinicians interpret why procedures with similar survival could have different short-term complication profiles. They were not systematically reviewed as outcomes in this umbrella review and should not be read as findings generated by it. Impact instruments deliver force in discrete pulses, whereas drill-based, hydraulic, piezoelectric, and osseodensification systems deliver force in other ways. These differences provide a plausible clinical context for reported discomfort or vertigo, but the included meta-analyses do not establish a causal link. The same caution applies to Wolff’s law [37,38], finite-element models, and the PASS principle [39]. These concepts may explain biological plausibility, but they do not replace direct comparative evidence.

### 4.3. Methodological Quality and Evidence Confidence

No included review achieved HIGH AMSTAR 2 confidence. Nine were MODERATE, 11 LOW, and 10 CRITICALLY LOW. The most common weaknesses were lack of prospective protocols, incomplete lists of excluded studies, and limited assessment of publication bias. We therefore gave greater weight to MODERATE reviews and used weaker reviews only as supporting context. This sensitivity check did not change the broad finding of high survival across treatment options. It did, however, reduce confidence in technique-specific complication claims because those claims often depended on sparse events or weaker reviews.

### 4.4. Primary-Study Overlap and Cluster-Level CCA

The pool-level Corrected Covered Area of 0.92% (slight overlap per Pieper et al. 2014 [7] thresholds) understates the dependence of specific synthesis claims on a small number of repeatedly re-analysed primary studies. The graftless osteotome-mediated/transcrestal sinus floor elevation cluster (seven meta-analyses; Moraschini 2017 [31], Rahate 2023 [30], Chen and Shi 2018 [11], Duan 2017 [9], Guo 2020 [17], Ye 2021 [19], Shi QH 2022 [21]) yielded a cluster-level CCA of 10.90% (high overlap), driven by a small set of RCTs (notably Nedir 2013/2016 and Si 2013/2016) that appear in five or more of the seven cluster meta-analyses. The short-implants-versus-sinus-floor-elevation cluster (eight meta-analyses after Phase 3 additions of Vetromilla 2021 [4] and Toledano 2022 [5]) yielded a cluster-level CCA of 6.8% (moderate overlap), reflecting shared reliance on the Thoma, Esposito, Felice, Guljé, Bechara, Bolle, Gastaldi, and Schincaglia RCT family, a base substantially expanded by Toledano’s 14-RCT pool.

The interpretive consequence is that synthesis claims regarding graftless approaches and short-implant equivalence are underwritten by a denser primary-study substrate than the pool-level CCA alone suggests. Replication is concentrated rather than distributed, meaning that any single high-quality primary study addressing one of these clusters could meaningfully shift the synthesis conclusions. Conversely, the perforation–survival, long-term LSFE, and technique-heterogeneity clusters showed lower overlap, reflecting independent primary-study substrates and therefore wider replication breadth.

### 4.5. Clinical Implications

The evidence applies to three different decisions. First, when residual bone height and implant stability allow it, short implants are a reasonable alternative to standard implants with sinus floor elevation; survival was similar in the relevant meta-analyses, while operative burden and complication profiles differed. Second, when sinus elevation is required, the choice between transcrestal and lateral access should be based on anatomy, required elevation, operator experience, and the different morbidity profiles reported in the literature. Third, when a transcrestal approach is selected, the present evidence does not support a firm ranking of osteotome, drill-based, hydraulic, piezoelectric, or osseodensification techniques. The reported BPPV and discomfort signal with osteotomes should be discussed with patients, but it remains based on few events. Clinical recommendation: choose the least invasive option that fits the anatomy and treatment goal, and do not use survival alone to distinguish procedures.

### 4.6. Limitations

Several limitations affect this review. Most included meta-analyses reported short- or medium-term outcomes. PROM evidence was sparse and used different instruments. The search was limited to English-language publications. The review was not prospectively registered in PROSPERO because the umbrella-review protocol was finalised after screening had begun; the Zenodo deposit improves transparency but does not replace prospective registration. We used narrative synthesis because the included reviews addressed different clinical questions and shared some primary studies. This prevented double counting but limited direct numerical comparison across review clusters. Rare complications, including BPPV and membrane perforation, were reported inconsistently and often without complete denominators or confidence intervals. Publication bias is therefore difficult to exclude, especially for rare events. Finally, the biomechanical discussion is an interpretation based on external biological and engineering concepts and is not a direct result of the umbrella review. Screening and extraction were verified by the team using complementary clinical and data-science expertise, rather than as a formal independent duplicate at every stage, which is a limitation.

## 5. Conclusions

This umbrella review found high implant survival across the main treatment options, but survival alone does not answer all clinical questions. Evidence should be interpreted in three steps: whether sinus elevation is needed, whether transcrestal or lateral access is appropriate, and which transcrestal technique fits the case. Reports of BPPV, discomfort, and other complications with osteotome-based procedures are suggestive but sparse. They do not establish a causal advantage for non-impact techniques or for any commercial device. Future reviews should keep these clinical questions separate, report raw complication counts and patient-reported outcomes, and avoid combining distinct techniques without clear subgroup data.

## Figures and Tables

**Table 1:** Overview of the 30 included meta-analyses on transcrestal sinus floor elevation, with comparator structure, primary outcome, and AMSTAR 2 confidence tier. References numbered as cited in the main text.

	First Author, Year [Ref.]	Journal	n	Comparator	Primary Outcome	AMSTAR 2
1	Duan 2017 [9]	J. Periodontol.	19	Graftless TSFA (single-arm)	Implant survival	CRITICALLY LOW
2	Geminiani 2017 [10]	Quintessence Int.	28	TSFA vs. LSFA—complications	Complication rates	CRITICALLY LOW
3	Chen & Shi 2018 [11]	J. Prosthodont.	12	OSFE: graft vs. no-graft	Implant survival; bone gain	LOW
4	Al-Moraissi 2018 [12]	Clin. Implant Dent. Relat. Res.	31	Membrane perforation vs. intact	Implant failure post-perforation	CRITICALLY LOW
5	Aludden 2018 [13]	Implant Dent.	9	LSFA with β-TCP only	Implant survival; bone formation	LOW
6	Antonoglou 2018 [14]	Int. J. Oral Maxillofac. Implants	21	SFA techniques (≥3-yr follow-up)	Implant survival	LOW
7	Al-Moraissi 2019 [15]	J. Oral Maxillofac. Surg.	36	Network MA: SFA techniques (RBH 4–8 mm)	Implant failure	LOW
8	Ravidà 2019 [16]	Implant Dent.	19	Short (≤6 mm) vs. long (≥10 mm) implants	Implant survival; complications	MODERATE
9	Guo 2020 [17]	Sci. Rep.	16	Non-grafted vs. PRF/PRP-grafted TSFE	Implant survival; bone gain	CRITICALLY LOW
10	Lin 2021 [18]	Int. J. Implant Dent.	12	TSFE + short implants (single-arm)	Implant survival	CRITICALLY LOW
11	Ye 2021 [19]	Int. J. Implant Dent.	27	OSFE without graft (single-arm)	Implant survival; bone gain	LOW
12	Tang 2022 [20]	Int. J. Implant Dent.	14	Short (≤8 mm) vs. standard (≥10 mm) implants after SFE	Implant survival; MBL	LOW
13	Shi QH 2022 [21]	Int. J. Oral Maxillofac. Implants	23	TSFA: graft vs. no-graft	Prognostic outcomes	LOW
14	Lee 2023 [22]	Int. J. Oral Maxillofac. Implants	38	Network MA: SFE techniques—perforation	Schneiderian membrane perforation	MODERATE
15	Potdukhe 2025 [23]	J. Prosthet. Dent.	11	Osseodensification vs. osteotome (TSFE)	Implant stability; bone height gain	CRITICALLY LOW
16	Shi S 2023 [24]	Clin. Oral Implants Res.	25	TSFA vs. LSFA at RBH ≤ 6 mm	Implant survival; perforation	MODERATE
17	Kadkhodazadeh 2024 [25]	Br. J. Oral Maxillofac. Surg.	17	TSFE techniques (RCTs)	Implant survival; complications incl. BPPV	MODERATE
18	Cobo-Vázquez 2025 [26]	Int. J. Implant Dent.	8	Osteotome vs. osseodensification (crestal SFE)	Bone height gain; complications	LOW
19	Starch-Jensen 2025 [27]	J. Oral Maxillofac. Res.	13	TSMEOD vs. LSFA/OMSFE	Bone gain; complications; PROMs	LOW
20	Menini 2025 [28]	Int. J. Oral Maxillofac. Implants	12	SFE techniques—PROMs	Patient-reported outcomes	LOW
21	Schiavo-Di Flaviano 2024 [29]	J. Clin. Med.	13	Membrane perforation vs. intact	Implant survival post-perforation	CRITICALLY LOW
22	Rahate 2023 [30]	J. Indian Soc. Periodontol.	9	OSFE simultaneous: graft vs. no-graft	Implant survival; bone height	CRITICALLY LOW
23	Moraschini 2017 [31]	Int. J. Oral Maxillofac. Surg.	11	SFE without graft + simultaneous implant	Implant survival	MODERATE
24	Shah 2022 [32]	J. Indian Prosthodont. Soc.	12	SFE techniques (survival comparison)	Implant survival	CRITICALLY LOW
25	Mester 2023 [33]	J. Pers. Med.	10	Short implants vs. SFE (RCTs ≥5 yr)	Implant survival; MBL	MODERATE
26	Yan 2019 [34]	BMJ Open	7	Short implants (≤6 mm) vs. SFE	Implant survival; complications	MODERATE
27	Wang M 2022 [35]	Materials (Basel)	6	Short implants vs. SFE (RCTs, ≥5 yr post-loading)	Implant survival	CRITICALLY LOW
28	Raghoebar 2019 [36]	J. Clin. Periodontol.	87	LSFE long-term (≥3 yr)	Implant survival	MODERATE
29	Vetromilla 2021 [4]	J. Prosthet. Dent.	7	Short implants vs. SFE (umbrella of 7 SRMAs)	Implant survival; MBL; complications	LOW
30	Toledano 2022 [5]	Clin. Oral Investig.	14	Short implants (≤6 mm) vs. SFE + standard implants (RCTs)	Implant survival; MBL	MODERATE

Note. TSFA = transcrestal sinus floor augmentation; LSFA = lateral sinus floor augmentation; OSFE = osteotome sinus floor elevation; SFE = sinus floor elevation; PRF = platelet-rich fibrin; PRP = platelet-rich plasma; β-TCP = beta-tricalcium phosphate; OD = osseodensification; TSMEOD = transcrestal modified expansion-osteotomy with drilling; OMSFE = osteotome-mediated sinus floor elevation; RBH = residual bone height; MBL = marginal bone loss; PROMs = patient-reported outcome measures; BPPV = benign paroxysmal positional vertigo; NMA = network meta-analysis. AMSTAR 2 confidence ratings reflect verbatim per-item assessment with second-pass verification.

**Table 2:** Operational classification of transcrestal techniques and summary of the available evidence.

Class	Operational Criterion	Examples	Survival	Complication and PROM Signal	Interpretive Limit
Impact osteotome	Mallet- or hand-driven osteotome with discrete axial impacts	Summers and related osteotome protocols	Generally 95–100%	BPPV and discomfort reported more often in some reviews; Cobo-Vázquez: 1/24 BPPV (4.17%) in an osteotome group	Sparse events; mixed study designs; no causal inference
Drill-based	Rotary preparation or reamer without mallet impact	TSFE reamer and controlled drill protocols	Generally 95–100%	Low perforation ranking in Lee 2023 NMA	Network ranking depends on the included primary studies
Hydraulic	Membrane elevation by fluid pressure	Hydraulic elevation devices	Generally 95–100%	Limited technique-specific complication data	No evidence that hydraulic is superior to other subclasses
Piezoelectric or ultrasonic	Oscillating tip used for bone preparation or elevation	Piezoelectric and ultrasonic systems	Generally 95–100%	Limited technique-specific data	Few direct comparisons
Osseodensification or expansion	Counter-rotating burs or expansion osteotomy without mallet impact	Densah and related expansion protocols	Generally 95–100%	Cobo-Vázquez: overall complications 2.78% OD vs. 14.32% OST; Starch-Jensen reported a BPPV difference	Small OD evidence base; differing denominators; no firm ranking
Mixed or unclear	Review combines more than one technique without separable subgroup data	Mixed TSFE reviews	Generally high	Not assigned to one mechanism class	Used only for the relevant clinical question, not for class comparison

Note. OD = osseodensification. Technique classes were assigned from the instrument and force-delivery description in each review. Multi-technique reviews were not forced into one class; results were assigned to a class only when the underlying subgroup or primary study could be identified. Otherwise, the review was labelled mixed. Full study-to-class mapping is provided in the Zenodo supplement.

**Table 3:** Descriptive evidence summary by outcome, with methodological anchors, overlap concerns, and limits on interpretation.

Outcome	Main Review-Level Evidence	Stronger AMSTAR 2 Anchors	Consistency	Overlap Concern	Main Limit on Interpretation
Implant survival	Broad evidence across the 30 reviews	Moraschini, Mester, Yan, Ravidà, Raghoebar, Toledano	Generally high across comparisons	Low pool-level overlap; moderate in some clusters	Different clinical questions and follow-up periods
Membrane perforation	Reported in several access and technique comparisons	Lee, Shi S, Kadkhodazadeh	Direction varies with comparison	Cluster-dependent	Often mixes transcrestal-vs-lateral and within-TSFE questions
BPPV	Reported in a small number of reviews	Kadkhodazadeh; limited support from Cobo-Vázquez and Starch-Jensen	Suggestive osteotome signal	Sparse events	Incomplete denominators and no stable pooled estimate
PROMs	One dedicated meta-analysis plus secondary reports	Menini (LOW); selected supportive reviews	Often favours less invasive procedures	Sparse and heterogeneous	Different instruments and time points
Short implants vs. SFE	Several RCT-based meta-analyses	Ravidà, Yan, Mester, Toledano	Similar survival; different burdens and complications	Moderate cluster overlap	Implant lengths and augmentation protocols differ

Note. This table is not a GRADE assessment. It summarises review-level quality, consistency, overlap, and reporting limits without assigning formal certainty levels.

## Data Availability

The supporting files for this umbrella review are openly available at Zenodo (https://doi.org/10.5281/zenodo.21432230, accessed on 18 July 2026) under a Creative Commons Attribution 4.0 International (CC-BY 4.0) licence. They include the search strategy, study-selection summary, included-review list, AMSTAR 2 ratings, CCA results and heatmap, source-checked complication and PROM summaries, technique mapping, and methodological decision notes. Published articles, copied article tables or figures, patient-level data, and internal technical files are not included.

## Supplementary Materials

The following supporting information can be downloaded at: https://www.mdpi.com/article/10.3390/dj14080483/s1, Table S1: PRISMA_2020_checklist.

## Abbreviations

The following abbreviations are used in this manuscript:

Abbreviation	Meaning
AMSTAR 2	A Measurement Tool to Assess Systematic Reviews 2
BPPV	Benign paroxysmal positional vertigo
CCA	Corrected Covered Area
LSFE/LSFA	Lateral sinus floor elevation/augmentation
MBL	Marginal bone loss
OD	Osseodensification
OMSFE	Osteotome-mediated sinus floor elevation
PROMs	Patient-reported outcome measures
RBH	Residual bone height
SFE	Sinus floor elevation
TSFE/TSFA	Transcrestal sinus floor elevation/augmentation

## Appendix A

**Table A1:** Examples of AMSTAR 2 critical-item ratings for five included reviews. Full item-level ratings and supporting quotations for all reviews are available at Zenodo. Y = Yes; PY = Partial Yes; N = No.

Review	Item 2 Protocol	Item 4 Search	Item 7 Excluded Studies	Item 9 Risk of Bias	Item 11 Meta-Analysis	Item 13 RoB in Interpretation	Item 15 Publication Bias	Overall
Ravidà 2019 [16]	PY	Y	PY	Y	Y	Y	Y	MODERATE
Lee 2023 [22]	Y	Y	PY	Y	Y	Y	Y	MODERATE
Kadkhodazadeh 2024 [25]	Y	Y	PY	Y	Y	Y	Y	MODERATE
Duan 2017 [9]	N	PY	N	PY	Y	PY	N	CRITICALLY LOW
Cobo-Vázquez 2025 [26]	Y	PY	N	Y	Y	PY	PY	LOW
